# Lysophosphatidylcholines and phosphatidylcholines as biomarkers for stroke recovery

**DOI:** 10.3389/fneur.2022.1047101

**Published:** 2022-12-15

**Authors:** Meiling Huang, Shaohang Xu, Mingchao Zhou, Jiao Luo, Fubing Zha, Linlin Shan, Qingqing Yang, Baojin Zhou, Yulong Wang

**Affiliations:** ^1^Department of Rehabilitation, Shenzhen Second People's Hospital, The First Affiliated Hospital of Shenzhen University, Shenzhen, Guangdong, China; ^2^Deepxomics., Ltd., Shenzhen, Guangdong, China; ^3^Department of Rehabilitation, Shenzhen Dapeng New District Nan'ao People's Hospital, Shenzhen, Guangdong, China

**Keywords:** lipidomics, mass spectrometry, stroke, lysophosphatidylcholines, biomarkers, stroke recovery

## Abstract

Stroke is a serious global public health issue, associated with severe disability and high mortality rates. Its early detection is challenging, and no effective biomarkers are available. To obtain a better understanding of stroke prevention, management, and recovery, we conducted lipidomic analyses to characterize plasma metabolic features. Lipid species were measured using an untargeted lipidomic analysis with liquid chromatography-tandem mass spectrometry. Sixty participants were recruited in this cohort study, including 20 healthy individuals and 40 patients with stroke. To investigate the association between lipids related to long-term functional recovery in stroke patients. The primary independent variable was activities of daily living (ADL) dependency upon admission to the stroke unit and at the 3-month follow-up appointment. ADL dependency was assessed using the Barthel Index. Eleven significantly altered lipid species between the stroke and healthy groups were detected and displayed in a hierarchically clustered heatmap. Acyl carnitine, triacylglycerol, and ceramides were detected as potential lipid markers. Regarding the association between lipid profiles and functional status of patients with stroke the results indicated, lysophosphatidylcholines (LPC) and phosphatidylcholines were closely associated with stroke recovery. LPC may contribute positively role in patient's rehabilitation process *via* an anti-inflammatory mechanism. Appropriate management or intervention for lipid levels is expected to lead to better clinical outcomes.

## 1. Introduction

Stroke is a life-threatening medical condition caused by inadequate blood supply to the brain. It is a rapidly progressive disease and the third leading cause of death worldwide among older adults (1, 2). Annually, more than two million people suffer from stroke in China (3). Stroke has become a major public health issue, resulting in increased medical expenditures (4). Although the survival rates have improved with modern treatment options, patients frequently experience permanent disability after stroke (5). Reliable interventions and accurate blood marker analyses are required to enhance the recovery process and reduce the disability rate in patients post-stroke.

Current stroke diagnostic techniques and pathologic measures have not yet determined the evolution of altered lipid metabolism in stroke and how it is associated with other stroke-related risk factors. The diagnosis of stroke relies mainly on several neuroimaging techniques, such as computed tomography (CT) scans, magnetic resonance imaging (MRI) scans, arteriography, and Doppler ultrasound. Existing techniques entail some limitations in discriminating the stroke type within the initial hours after the event and may even fail to identify the association with future stroke recovery (6–8). Investigation and understanding of the pathophysiological mechanisms of stroke can contribute to the promotion of secondary prevention and patient management. Therefore, diagnostic biomarkers that can determine the pathophysiological changes in multiple organs need to be explored as they may explain changes related to acute stroke, including brain injury, systemic response, and even stroke progression.

Lipid metabolism disorders are controllable risk factors that contribute to stroke development (9). Traditional lipid markers, including total cholesterol, triglycerides, low-density lipoprotein-cholesterol (LDL-C), and high-density lipoprotein-cholesterol (HDL-C), are often used to evaluate stroke risk as they are altered in stroke as a result of dysfunctional lipid and lipoprotein metabolism (10). However, they cannot help discriminate how lipid dysfunction occurs at the molecular level or identify metabolites associated with stroke prognosis in an early stage. It is critical to identify novel biomarkers that discriminate, diagnose, and classify stroke quickly and accurately.

Lipids have emerged as potential biomarkers for various diseases. Since lipids serve as structural components of cell membranes, signaling mediators, cellular barriers, and energy depots, they can reflect the pathological or physiological status of metabolic disorders (11–13). In the central nervous system, lipids and lipid mediators play a key role in maintaining structure and function of brain tissue (10). To a large extent, they can affect stroke patient's outcomes and recovery. A better understanding of the functional activities of distinct lipid molecules provides an opportunity to better understand the roles of lipids in the origin of stroke, and to identify novel lipid biomarkers and therapeutic targets. Novel explorations in mass spectrometry technologies for lipidomics have enabled the simultaneous untargeted detection and quantification of thousands of lipids, which can substantially help in diagnosing and understanding diseases (14, 15).

In this study, we aimed to investigate the lipids associated with physiological responses or development of stroke and which can provide explanation to the recovery of stroke. By integrating liquid chromatography–tandem mass spectrometry (LC–MS) techniques and advanced bioinformatic analyses, we applied untargeted lipidomic profiling to screen for detectable lipids in the plasma of a cohort of patients with stroke, using healthy participants as controls.

## 2. Materials and methods

### 2.1. Patient enrolments

This study recruited 40 patients with acute ischemic stroke hospitalized in neurology department of Shenzhen Second People's Hospital from January 2021 to August 2021, within 48 h of experiencing stroke symptoms. Twenty healthy people with non-cardiovascular and cerebrovascular diseases in the same physical examination center were randomly selected as non-stroke control group. Inpatients who met the inclusion criteria were selected by convenient sampling and they were recruited in the study through in-hospital poster advertising.

The study was conducted in accordance with the Declaration of Helsinki. All study protocols and methods were approved by the Ethics Committee of Shen-zhen Second People's Hospital (No. 20200601044-FS01) and registered in the Chinese Clinical Trial Registry (ChiCTR2000035352). Written consent was obtained from all participants.

The inclusion criteria for patients with stroke were as follows: (1) diagnosed with acute cerebral stroke *via* CT or magnetic resonance imaging; (2) aged 18 years and above; (3) admitted within 48 h of stroke onset; and (4) with no history of cerebrovascular disease.

The exclusion criteria for patients with stroke were as follows: (1) hemorrhagic stroke; (2) spinal cord injury, motor neuron disease, or Parkinson's disease; (3) autism, Alzheimer's disease, or developmental delay; (4) unconsciousness; (5) intravenous thrombolytic therapy; and (6) inability to cooperate with the evaluation.

Twenty healthy controls were recruited using the following inclusion criteria: (1) aged 18 years or older, (2) no history of cerebrovascular disease, and (3) no sign of stroke based on CT or magnetic resonance imaging examination. The exclusion criteria were the same for both groups.

### 2.2. Clinical data collection

All data were collected in interviews in person by professional healthcare workers, such as doctors and physiotherapists, who were trained for this study. Baseline characteristics, including name, sex, stroke type, and disease duration, were documented. Participant's weight and height were obtained and their body mass index (BMI; kg/m^2^) was then calculated. Clinical biochemical indicators were collected from patients with stroke upon hospitalization and from the medical examinations of healthy participants.

The ability to perform activities of daily living (ADL) of 40 patients with stroke was evaluated using the Barthel Index (BI) (16) on admission and in the follow-up appointment at 3 months post-stroke. Recovery was assessed at 3 months using the BI score. The BI scoring method was based on a previous study (17). The BI score ranges from 0 to 100, 0 indicates complete dependence for ADL and 100 indicates complete independence for ADL. The study participants with BI scores ≤90 were considered to have an undesirable recovery outcome and determined to be dependent (18). To minimize bias, two professional physiotherapists were selected from our rehabilitation department and trained for this study to evaluate the BI scores of all participants.

### 2.3. Sample preparation and lipid extraction

Plasma collection was conducted by venipuncture into ethylenediaminetetraacetic acid tubes upon hospitalization for non-treatment with tissue plasminogen activator delivery or mechanical thrombectomy. Before the experiments, the samples were thawed at 4°C until no ice was observed in the tubes. Lipid extraction was performed in accordance with a previously reported protocol (19). Briefly, 40 μl of serum was extracted with 120 μl of precooled isopropanol (IPA) and vortexed for 1 min. After incubation at ambient temperature for 10 min, the mixture was stored overnight in a refrigerator at −80°C to facilitate protein precipitation. The samples were centrifuged for 20 min at 16,000 × *g*, and the supernatant was kept at −80°C until the LC–MS analysis. Pooled quality control (QC) samples were prepared to evaluate the LC–MS system conditions by mixing equal volumes of all samples.

### 2.4. Lipid detection with LC–MS

The extracted lipids were separated using an ACQUITY UPLC BEH-C18 (2.1 × 100 mm, 1.7 μm, Waters, Milford MA, USA) on a Vanquish Flex system (Thermo Fisher Scientific, MA, USA) and emitted into a Q-Exative mass spectrometer (Thermo Fisher Scientific). A flow rate of 0.3 ml/min was applied to mobile phase A, which included 10 mM ammonium formate and 0.1% formic acid (ACN:H_2_O = 60:40, v/v), and mobile phase B, which contained 10 mM ammonium formate and 0.1% formic acid (IPA:ACN = 90:10, v/v). The initial elution began at 30% B and was quickly evaluated using a linear gradient to 60% B for the first 3.5 min, followed by an increase to 100% B within 5 min. Finally, B was restored to 30% over the next 0.1 min and equilibrated for 1.4 min before the subsequent injection. The Thermo Q-Exactive was operated with the following parameters: spray voltage 4 kV (positive) and −4 kV (negative). For both ionization modes, the sheath gas and aux gas were separately maintained at 35 and 10 arbitrary units, while the capillary temperature and the heater temperature were 320 and 350°C, respectively. The MS/MS data was acquired by data dependent method and top 3 abundant ions were used for fragmentation. The normalized collision energy (NCE) was set 15, 30, and 45 eV, respectively.

### 2.5. Data preprocessing, quantification, and identification

ProteoWizard was used to convert raw data to mzXML format (20). Based on the R environment, the metaX toolbox integrated with the XCMS package read and processed MS data, including peak picking, peak grouping, retention time alignment, and second peak grouping (21, 22). Ion features were detected and extracted according to retention time (RT) and *m*/*z* using the XCMS package. A three-dimensional matrix containing randomly assigned peak indices (retention time-*m*/*z* pairs), sample names (observations), and ion area was generated. Next, the raw peak areas from the ion features were processed using the MetaX package. Observations found in <50% of the QC samples or 80% of the biological samples were excluded. The *K*-nearest neighbor imputation method was used to handle missing values. A probabilistic quotient normalization algorithm was introduced to normalize the data for all samples (23). Quality control-based robust LOESS signal correction was fitted to the QC data with respect to the order of injection to minimize the signal intensity drift over time (24). A partial least squares discriminant (PLS-DA) model was built to calculate the variable importance in projection (VIP) score for each lipid feature.

All features were searched against MS2 libraries using MSDIAL (version 4.0) (25). Mass tolerance was configured as 0.01 for MS1 and 0.05 for MS2 for both positive and negative modes. The minimum cutoff score for identification was set at 0.7. The MS2 identification entries from all files were integrated with the features quantified by MS1 *m*/*z* and RT using an in-house script.

### 2.6. Statistical exploration and downstream processing

All statistical analyses were performed using the R software for statistical computing and graphics (version 4.1.0; R Foundation, Vienna, Austria). To explore potential indicators of stroke recovery, the Shapiro-Wilk test and Wilcoxon rank-sum test was used to verify the assumptions of normality and to compare patients at the time of admission and those at the 3-month follow-up appointment, respectively. Normalized expression values were calculated for each row (each lipid feature) using *Z*-scores. A hierarchical clustering heat map was then generated to illustrate the overall characteristics of markers or lipid species using the ComplexHeatmap package (version 2.8.0) (26). The G-Power (27) was ued to estimate the sample sizes for each group based on given power value, effect sizes and α-levels. Two boxplots supplemented with corresponding *p*-values for particular lipid species were visualized to highlight the significance of the function from the ggpubr package (version 0.4.0).

The lipid co-expression network was generated by calculating the Pearson correlation coefficient of each lipid pair using the igraph package (version 1.2.11) (28). Lipids were depicted as colored vertices. The edge list consisted of pairs with a correlation value >0.85. Edges with absolute values below the cutoff were deleted from the network. The absolute value of the correlation determined the thickness of each edge, and the size of each vertex represented betweenness centrality. The network substructure was generated by calculating community membership modules using the Louvain method (29).

## 3. Results

### 3.1. Participant characteristics

**Figure 1:**
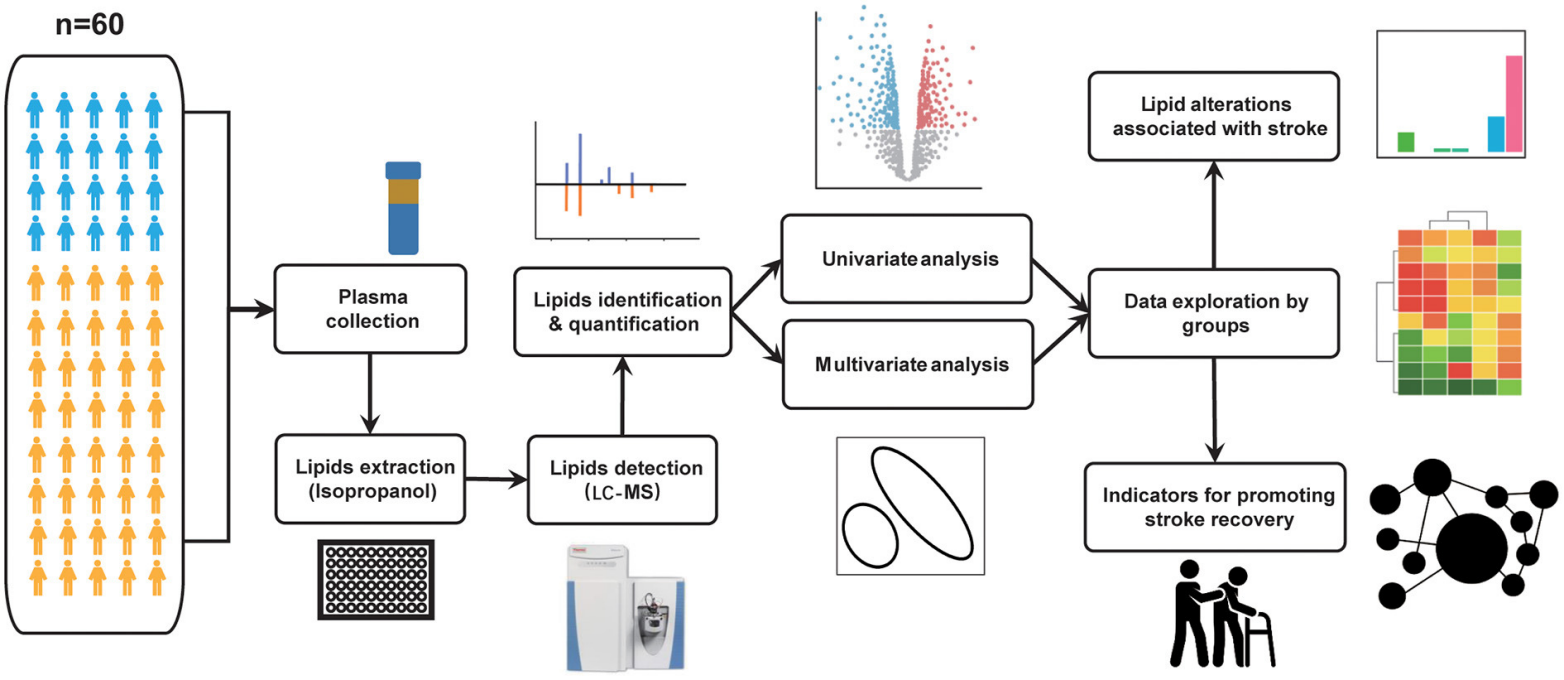
Flowchart illustrating the comprehensive framework of this study. A total of 60 subjects were recruited for this study, including 20 healthy and 40 acute stroke individuals. Plasma samples were collected from all study participants and pre-treated as described in the methods section. Using the untargeted profiling strategies by LC-MS platform, lipid features were identified and quantified from plasma samples. Multivariate and univariate analyses are used for data quality assessment and for exploring altered lipid molecular species among groups. Following that, we explored the data on two levels. (1) Based on the three groups mentioned above, we used the T test or Wilcoxon rank-sum test to identify differential lipid species in each group. (2) Based on the Barthel Index, we re-divided the patients into good and poor recovery groups. Potential indicators were detected based on the Wilcoxon rank-sum test. In addition, we built a network to investigate the correlations or interactions between pairs of lipid molecules.

The experimental design is illustrated in Figure 1. Sixty participants were recruited in this cohort study including 20 healthy participants and 40 stroke patients. Demographic information and clinical biochemical indicator results are presented in Table 1. As indicated in Table 1, patients with stroke had a slightly elevated BMI compared to that of healthy participants. Patients with stroke had significantly higher triglyceride but lower HDL levels compared with those from the non-stroke group (all *p* < 0.05). Moreover, the total protein level was significantly lower, whereas the fibronectin level was significantly higher in the stroke group compared to that in the non-stroke group (all *p* < 0.01). We also found significantly decreased albumin levels (*p* < 0.01) in patients with acute stroke compared to those observed in healthy participants.

**Table 1:** Demographic information.

**Clinical parameter**	**Total (*n* = 60)**	**Non-stroke (*n* = 20)**	**Stoke (*n* = 40)**	***p*-Value**
Male	34 (56.7)	10 (50)	24 (60)	0.645
BMI	23.70 ± 3.57	22.34 ± 3.18	24.33 ± 3.61	0.058
Lipid levels in mg/dl				
Total cholesterol	4.79 ± 1.05	4.93 ± 0.60	4.748 ± 1.17	0.484
Triglyceride	1.09 (0.77, 1.62)	0.72 (0.51, 1.12)	1.16 (0.91, 1.72)	0.016
HDL-C	1.15 ± 0.35	1.49 ± 0.36	1.04 ± 0.27	0.001
LDL-C	2.94 ± 0.86	2.79 ± 0.51	2.99 ± 0.95	0.360
Liver function in g/L				
Total protein	70.49 ± 6.006	75.956 ± 4.597	68.85 ± 5.42	0.001
Albumin	41.6 (39.05, 45.25)	48.60 (46.10, 49.60)	40.9 (38.425, 43.25)	0.001
tPA administration (%)	NA	NA	NA	
Stroke recovery scoring				
BI score at baseline		NA	68.88 ± 26.57	
BI score at 3 months		NA	83.13 ± 30.35	

Values are shown as n (%), mean ± SD, or median (25th and 75th percentiles). Chi-square test was utilized for the testing of categorical variables. And t-tests or Wilcoxon rank-sum tests were used to test the statistical significance of continuous variables based on data distribution.

BMI, body mass in dex; HDL-C, high-density lipoprotein cholesterol; LDL-C, low density lipoprotein-cholesterol; tPA, tissue plasminogen activator; BI, Barthel Index.

### 3.2. QC of lipidome analysis

**Figure 2:**
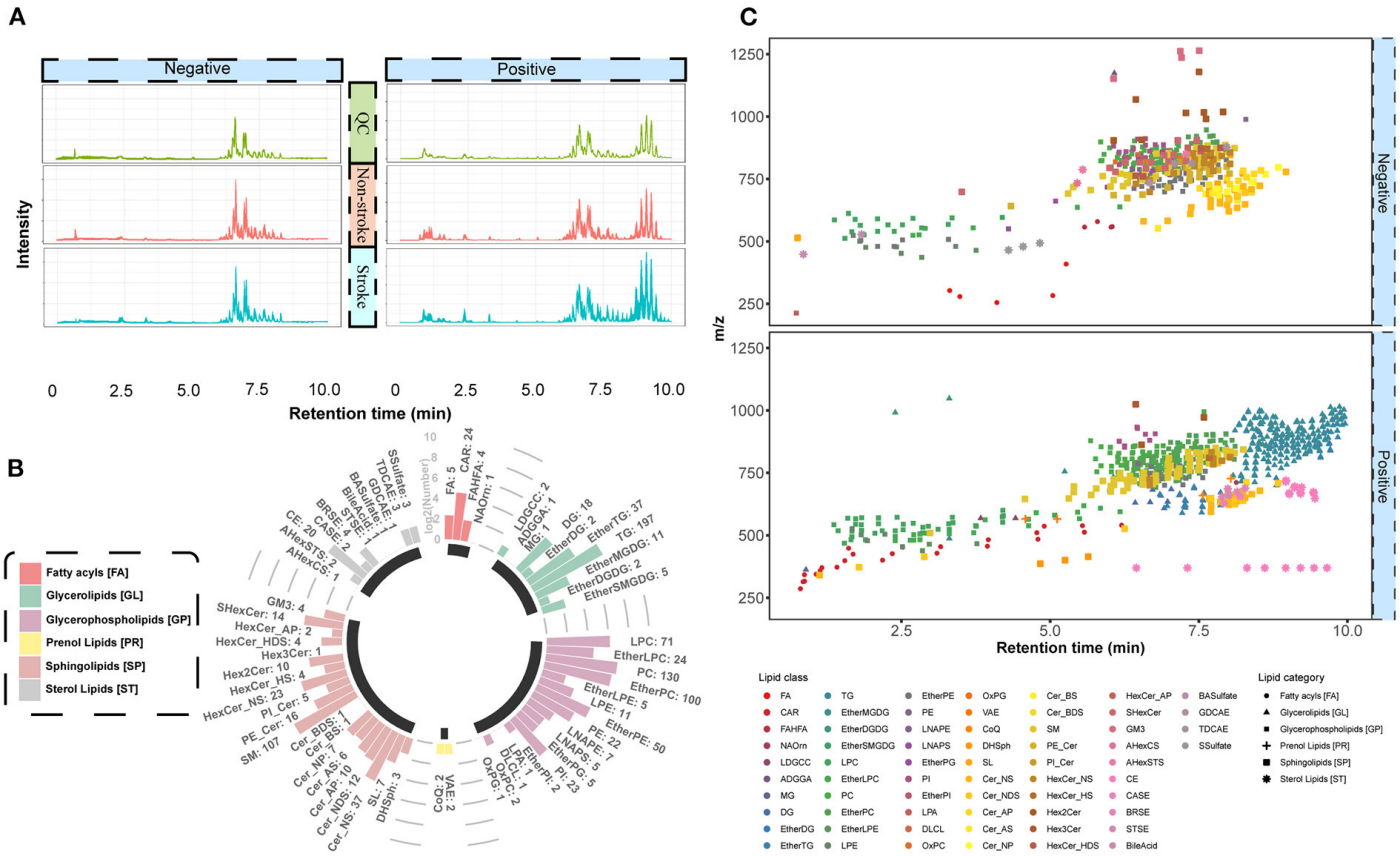
Quality assessment for lipids. **(A)** Total ion chromatogram of all samples. Negative and positive ion modes are illustrated in the left and right panels, respectively. Four colors correspond to four types of samples, including QC, non-stroke, and stroke groups. The *X*-axis displays the amount of time (min) for the ions to pass through the column. The *Y*-axis displays the peak area based on the number of ion counts taken by the mass spectrometer detector at the retention point. **(B)** Circular bar plot for identified lipids. The lipid species fall into six main categories with different colors. The numbers next to the lipid species name represents the number of lipid features that have been identified. **(C)** Spatial distribution and elution order for all detected lipids, plotted against their respective retention time (min) and *m*/*z*. The color of the peak spots indicates the lipid species, and the shape of the peak spots indicates six main lipid categories.

After analyzing the raw MS data, the untargeted lipidomic analysis generated 18,123 features in positive ion mode and 8,210 features in negative ion mode. All spectra were measured in the *m*/*z* range of 100–1,500. The distribution curves from the positive and negative modes revealed an appropriate complementarity between the two modes (Figure 2A). After peak matching, alignment, and missing value imputation, we identified 718 and 400 features through similarity measures of the MS/MS spectral library comparison in the positive ion mode and negative ion mode, respectively. The identified lipids belong to six major categories and 64 lipid species (Figure 2B). Among these, glycerolipids, glycerophospholipids, and sphingolipids were the three major lipid classes that covered the greatest number of identified lipids in our data. To promote confidence in lipid detection, we provided a peak spot graphic to illustrate the spatial distribution of all detected lipid species in two-dimensional spaces of RT and *m*/*z* (Figure 2C). Appropriate identification was determined by the correct fraction (lipid species) and matched with the correct mass and elution order. Specifically, lipid species with mass ranging from 250 to 600 Da eluted earlier than 5 min; lipid species with mass ranging from 600 to 800 Da fell into a specific range of RT of 5–8 min; and lipid species with mass ranges of more than 800 Da eluted after 8–10 min.

### 3.3. Differential lipids analysis

**Figure 3:**
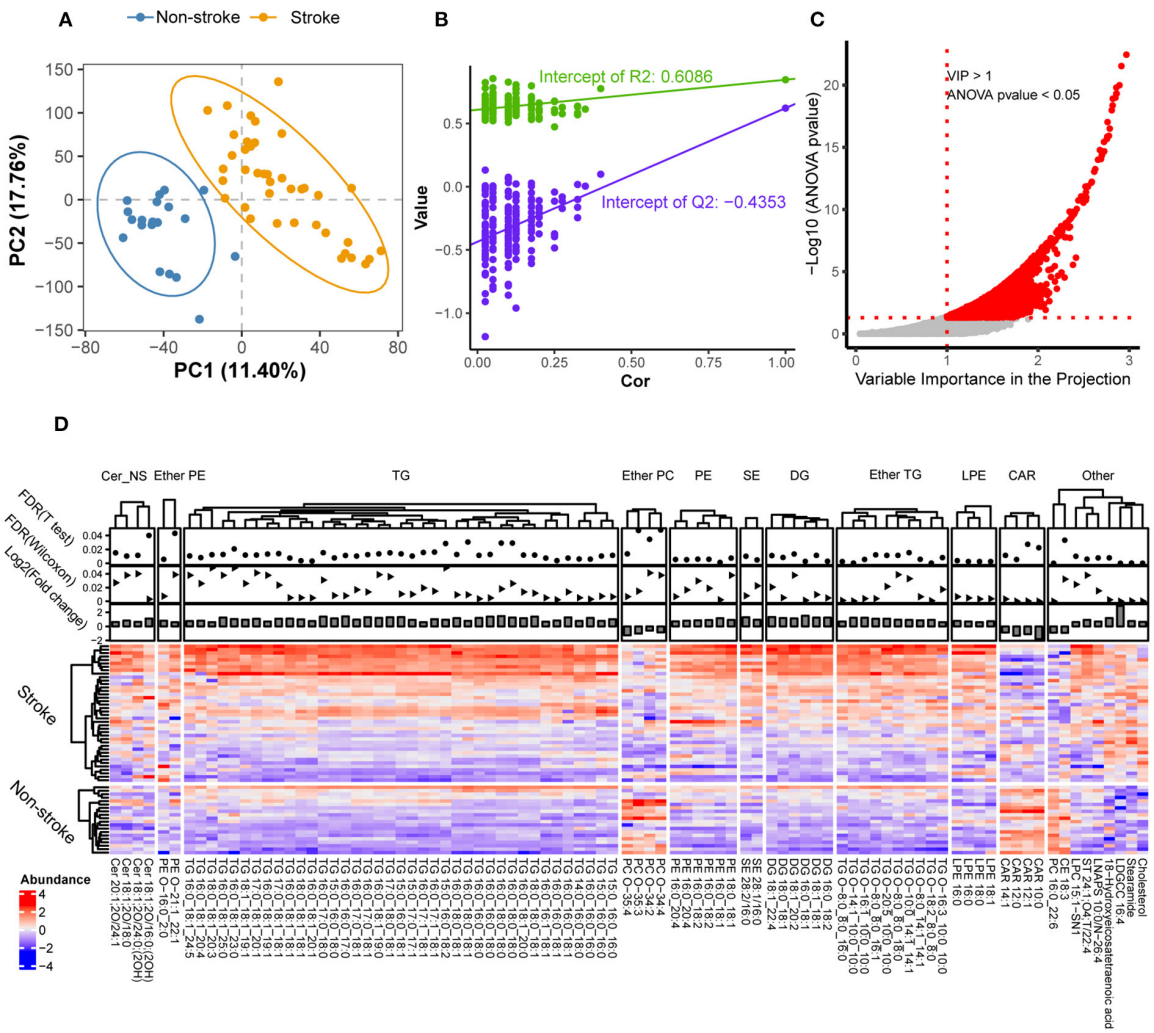
Significant differences in lipids between stroke and non-stroke groups. **(A)** PLS-DA score plots of the two groups with different colors. The first two principal components of PC1 and PC2 were illustrated on the *X*-axis and *Y*-axis, respectively. **(B)** Cross-validation plot from 200 cycles permutation tests for performance evaluation of the PLS-DA model. The green and purple dots represents the intercept values of R2 and Q2 from the permutation test, respectively. **(C)** Scatter plot of VIP vs. –log10(*p*-values) for significantly changed lipid species. Two red dotted lines represent the cut-offs for VIP > 1 and *p*-value < 0.05, respectively. **(D)** Significantly altered lipids are illustrated by a heatmap. Red indicate high abundance and blue indicate low abundance. *Z*-score method was used to calculate the normalized abundances for each row. Each column represents a specific lipid feature. And they were divided into 11 lipid classes according to their headgroups. A solid circle and triangle point graphs provided the false discovery rate of differentially expressed metabolic features with *T*-test and Wilcoxon rank-sum test, respectively. The bar charts provided the log_2_ fold change of differentially expressed metabolic features. Cer, ceramides; ether PE, ether-linked phosphatidylethanolamine; TG, triacylglycerol; ether PC, ether-linked PC; PE, phosphatidylethanolamine; SE, steryl esters; DG, diradylglycerols; ether TG, ether-linked TG; LPE, lysophosphatidylethanolamine; CAR, acyl carnitine.

All features were subjected to PLS-DA to detect lipid-driving group separations, and those with a high VIP score (≥1.0) were further assessed using statistical tests to identify dysregulated lipids associated with stroke development. As shown in Figure 3A, the score plot exhibited a clear separation trend between the stroke and non-stroke groups. Cross-validation was applied to the OPLS-DA model to check whether the model was overfitting. The intercept values of R2 and Q2 from the permutation test were 0.6086 and **–**0.4353, respectively, indicating there was no overfitting in the model (Figure 3B). Features with VIP ≥ 1 and an analysis of variance *p*-value ≤0.05 were selected as significantly differentially abundant lipids (Figure 3C).

Next, we compared the feature abundances for each group using heatmap visualization (Figure 3D). The dot plot shows the false discovery rate, and the bar plot shows the fold change for all differentially expressed lipid features at the top of the graph.

Lipids were considered differentially expressed and retained based on the following criteria: fold change of ≥1.5 at *t*-test and Wilcoxon rank-sum test false discovery rate ≤0.05. In total, 90 significantly differential lipid features were detected, which were further divided into 11 lipid groups. In particular, eight lipid species that were upregulated in the stroke group were detected: ceramides (Cer_NS), phosphatidylethanolamine, ether-linked phosphatidylethanolamine, triacylglycerol (TG), ether-linked TG (ether TG), steryl esters, diradylglycerols, and lysophosphatidylethanolamine. Two lipid species that were downregulated in the stroke group were detected: acyl carnitine (CAR) and ether-linked phosphatidylcholines (PC). In summary, these results provided initial evidence that lipid alterations may play a role in stroke pathophysiology.

### 3.4. Biomarkers for stroke recovery based on BI

**Figure 4:**
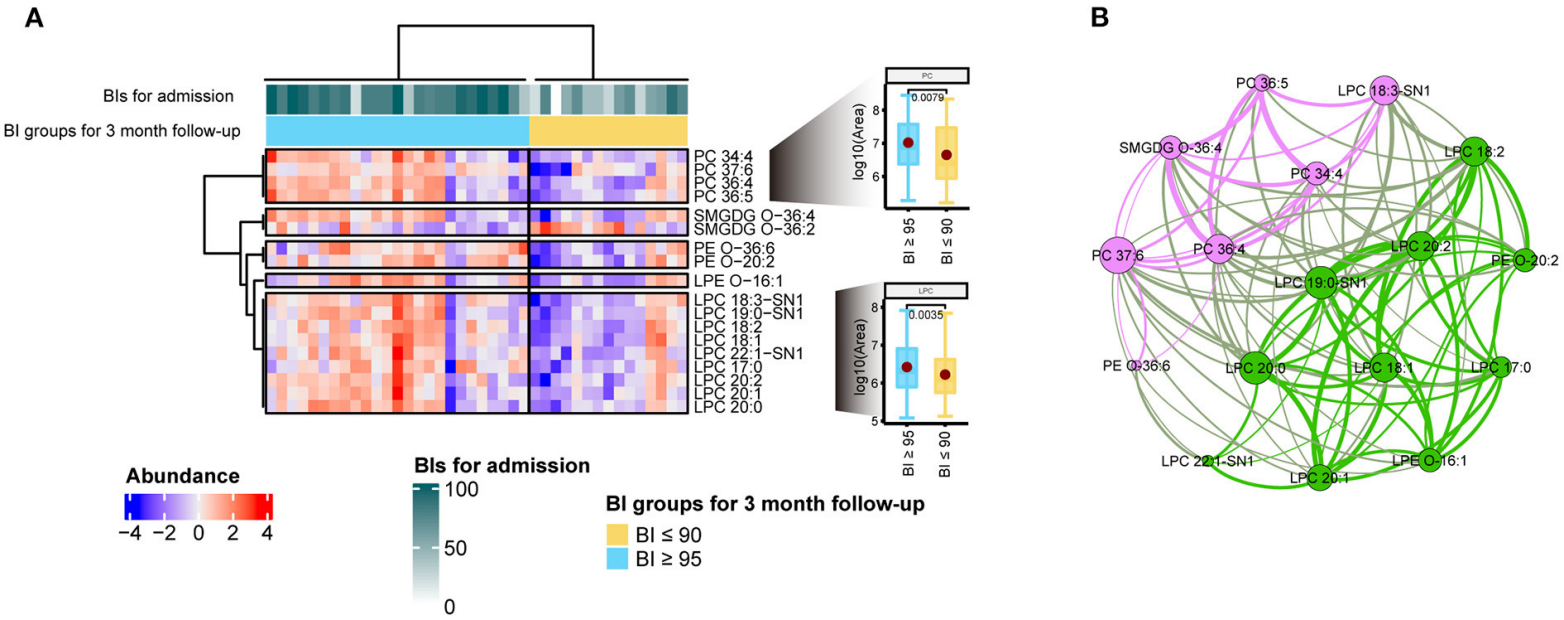
Indicators for stroke rehabilitation. **(A)** Heatmap of the 18 significantly changed lipid features associated with stroke rehabilitation. All features were split into five groups by lipid species. Two box plots provided the comparisons between good and poor recovery groups for LPC and PC species, respectively. The annotations for each individual were displayed as three-colored tracks on top of the heatmap. *Z*-score normalization was used to compare the lipid abundance over samples. **(B)** Correlation-based lipid network. Each node represents a lipid feature, and the edge represents the correlation between lipids. All pairwise correlation values for each node were calculated under the R environment. Two modularity classes were identified by the Louvain method of community detection and coded by the pink and green colors in the network, respectively. The importance or essentiality of a lipid node in the network is determined by its betweenness score. A node with a larger size indicates a higher betweenness score.

To investigate which biomarkers are related to stroke recovery, the BI scores of 40 patients with stroke upon admission and at the 3-month follow-up appointment were collected. As suggested by previous studies (18, 30, 31), these 40 patients were divided with stroke into two groups: ADL independent with a BI score ≥95 and ADL dependent with a BI score ≤90 using the BI score from the 3-month follow-up appointment. Next, a hierarchically clustered heatmap was constructed to present the lipids that were closely linked to these two groups. As shown in Figure 4, all individuals could be roughly divided into two predefined categories. The annotations for each individual are displayed as three-colored tracks on top of the heatmap. Moreover, significantly altered lipids were mainly involved in two lipid species: lysophosphatidylcholines (LPC) and PC. The Wilcoxon rank-sum test, accompanied by a boxplot, was used to validate the alternative hypothesis that lipid abundances were significantly different between the two groups. Furthermore, the lipid-related network was constructed based on the correlation coefficients of each lipid, suggesting that two major species, LPC and PC, play a dominant role in the recovery of patients with stroke.

## 4. Discussion

In this study, the lipid markers CAR, TG, and Cer_NS were detected in patients with stroke. Notably, Cer_NS expression levels were upregulated in the stroke group. TG expression levels were increased, while CAR expression levels were decreased in the stroke group. These results provide initial evidence that lipid changes were related to stroke pathogenesis. Furthermore, to improve the recovery process and reduce the disability rate in patients post-stroke, we explored the association between lipid profiles and functional status of patients with stroke and found that some LPC and PC were closely associated with stroke recovery.

Dyslipidemia is strongly associated with the pathogenesis of stroke, particularly ischemic stroke. Traditional lipid parameters, such as elevated concentrations of TC, TG, LDL-C, and reduced HDL-C concentrations, are considered as important risk factors and predictors of cardiovascular disease, especially for stroke (32, 33).

Tanne et al. (34) noted that excess triglycerides can increase the risk of stroke. Their research further demonstrated that individuals with low HDL levels were more likely to experience a stroke (34). These results are consistent with those of our research, and we found that several TGs showed increased expression in patients with stroke (Figure 3). Congruently, we observed a positively correlated with TG levels and negatively correlated with HDL levels in the clinical and pathological features dataset (Table 1). Therefore, these data provide further molecular evidence supporting dyslipidemia as one of the most important risk factors for stroke. Dyslipidemia is a modifiable risk factor for stroke. Appropriate management can be considered a key step for subsequent stroke recovery.

Moreover, Cer_NS expression levels were upregulated in the stroke group. Cer_NS is a bioactive sphingolipid with cellular signaling and secondary messenger capabilities, and contributes to various physiological processes, such as cell proliferation, senescence, adhesion, differentiation, and apoptosis (35, 36). A study suggested that focal cerebral ischemia increases the levels of Cer_NS in older adults and induces inflammatory processes (37, 38). Recently, a significant number of prospective cohort studies have found that several plasma Cer_NS can be used as reliable predictors to evaluate the severity or risk of stroke in patients upon admission (39–41). Here, Cer_NS was also significantly increased in the stroke group, suggesting that elevation of Cer_NS levels had a negative impact on patients with stroke.

Conversely, CAR was decreased in the stroke group. CAR has been indicated as an endogenous compound that is responsible for energy production through mitochondrial metabolic pathway of free fatty acids (42). Due to its capacity to support brain cells and promote alertness, CAR is commonly utilized as a brain booster by individuals of all ages. CAR has also been found toalso reportedly exerts neuroprotective effects against stroke by enhancing mitochondrial function and decreasing inflammation (43, 44). Several CARs were significantly lower in the stroke group than in the healthy group. This evidence suggests that CAR supplementation may have beneficial effects on stroke rehabilitation by enhancing functional recovery.

To investigate the association between lipid profile components and functional recovery after stroke, we screened for that were considerably different within two groups and we found 18 of these features (Figure 4). These lipids can be divided into two main lipid species, PC and LPC. LPC species are an important category of bioactive compounds linked to inflammatory disorders (45); and participates in many signaling pathways associated with oxidative stress and inflammatory processes (46–48). The generation of reactive oxygen species are closely associated with the pathogenesis of acute ischemic stroke, inducing brain injury, and worsening neurological prognosis (49). Inflammatory responses are involved in all stages of the ischemic cascade in stroke, from early adverse events to late regenerative processes after stroke (50). Anti-inflammatory therapy is considered one of the most promising methods for promoting neurorehabilitation after a stroke (51). Moreover, previous *in vivo* and *in vitro* experiments have demonstrated that LPC has a potential protective effect in preventing ischemic injury or neuronal death in patients with stroke (52). Therefore, we speculated that plasma LPC levels can serve as a potential biomarker for stroke care and recovery.

Similar to LPC, PC exhibited the same trend, with higher intensities in the BI ≥95 group. LPC is primarily produced by the turnover of PC and can be recycled to PC with the help of LPC acyltransferase (53). Furthermore, most lipids from these two species have mono- or polyunsaturated bonds in their carbon chains. Our network analysis also revealed that they are closely associated with each other. This evidence indicates that LPC and PC can be implemented as effective biomarkers for stroke recovery. Furthermore, anti-inflammatory interventions during stroke rehabilitation are expected to be an important means of reducing brain injury and achieving a better quality of life.

Our study had some limitations. First, this was only a small patient cohort study in China and further research with broader populations is needed to validate our findings. Second, targeted metabolomics technology in other samples is required to confirm the changes in lipid signatures. Finally, it is necessary to confirm the level of endogenous LPC and determine the clinical therapeutic value of LPC in patients with stroke or animal models.

## 5. Conclusions

We used LC–MS to analyze the plasma lipidomic profiles of individuals with stroke. Several lipid species, including CAR, TG, and Cer_NS, exhibited significant differences in their quantity. LPC and PC were found to be closely associated with stroke recovery. These lipid species have been implicated in the inflammatory response, antioxidative effects, and cell membrane protection. As a result, our findings might provide helpful information for achieving better clinical outcomes through management or intervention of lipid levels during the rehabilitation process of patients with stoke.

## Data availability statement

The datasets presented in this study can be found in online repositories. The name of the repository and accession number can be found at: National Omics Data Encyclopedia (NODE), https://www.biosino.org/node/search, OEP003223.

## Ethics statement

All study protocols and methods were approved by the Ethics Committee of Shen-zhen Second People's Hospital (No. 82 20200601044-FS01). The patients/participants provided their written informed consent to participate in this study.

## Author contributions

Funding acquisition and conceptualization: YW and MH. Study design: YW, MH, and MZ. Project administration, data acquisition, and writing original draft: MH. Sample collection: QY. Data analysis and/or interpretation: SX and BZ. Investigation: JL, LS, and FZ. Writing—review and editing: MH, YW, and SX. All authors have reviewed, approved, read, and agreed to the published version of the manuscript.
