# Supplementary material for: Lysophosphatidylcholines and phosphatidylcholines as biomarkers for stroke recovery
Source: Front Neurol. 2022 Dec 15;13:1047101. doi: 10.3389/fneur.2022.1047101 (PMC9797831; doi:10.3389/fneur.2022.1047101)
Supplement: Supplementary file 1 [file Image_1.pdf]

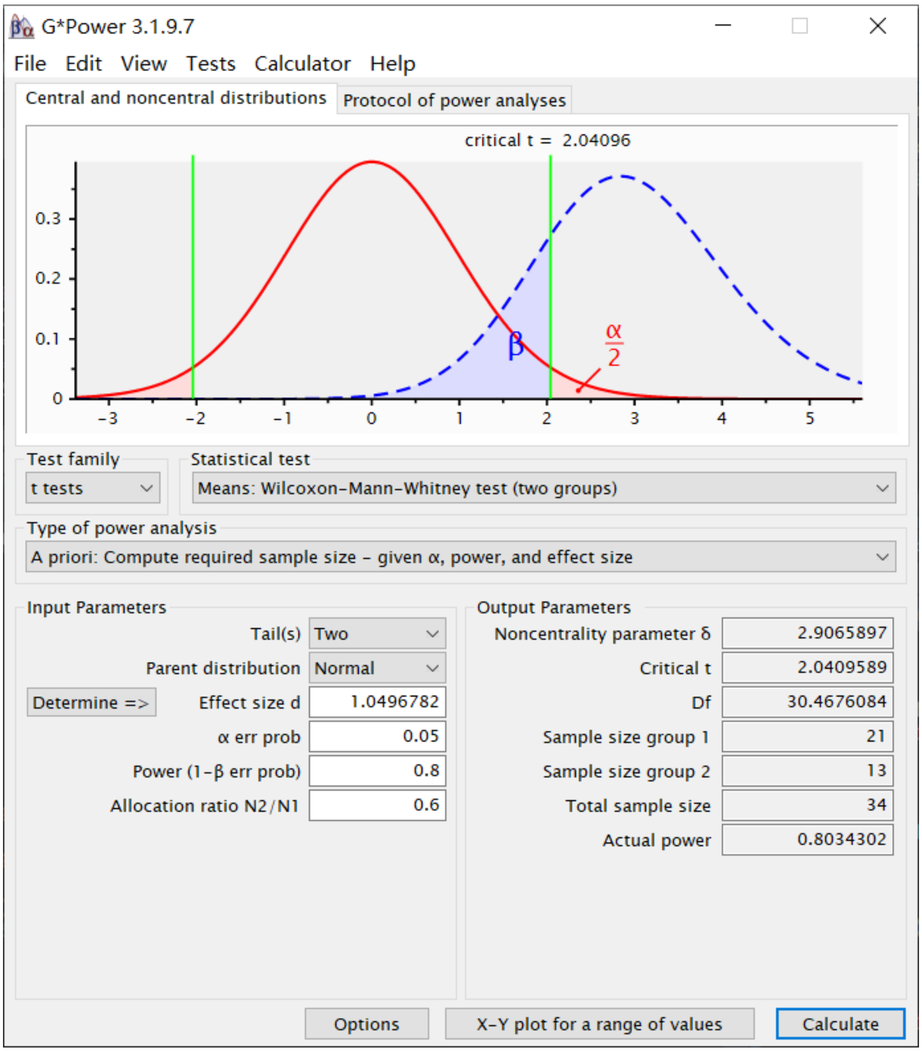

Figure S1. The G-power estimation of sample size for Barthel>90 and Barthel<90 group based on given power value, effect sizes and α-levels
